# A full Bayesian hierarchical mixture model for the variance of gene differential expression

**DOI:** 10.1186/1471-2105-8-124

**Published:** 2007-04-17

**Authors:** Samuel OM Manda, Rebecca E Walls, Mark S Gilthorpe

**Affiliations:** 1 Biostatistics Unit, Centre for Epidemiology and Biostatistics, Leeds, LS2 9LN, UK; 2 Department of Statistics, University of Leeds, Leeds, UK

## Abstract

**Background:**

In many laboratory-based high throughput microarray experiments, there are very few replicates of gene expression levels. Thus, estimates of gene variances are inaccurate. Visual inspection of graphical summaries of these data usually reveals that heteroscedasticity is present, and the standard approach to address this is to take a log_2 _transformation. In such circumstances, it is then common to assume that gene variability is constant when an analysis of these data is undertaken. However, this is perhaps too stringent an assumption. More careful inspection reveals that the simple log_2 _transformation does not remove the problem of heteroscedasticity. An alternative strategy is to assume independent gene-specific variances; although again this is problematic as variance estimates based on few replications are highly unstable. More meaningful and reliable comparisons of gene expression might be achieved, for different conditions or different tissue samples, where the test statistics are based on accurate estimates of gene variability; a crucial step in the identification of differentially expressed genes.

**Results:**

We propose a Bayesian mixture model, which classifies genes according to similarity in their variance. The result is that genes in the same latent class share the similar variance, estimated from a larger number of replicates than purely those per gene, i.e. the total of all replicates of all genes in the same latent class. An example dataset, consisting of 9216 genes with four replicates per condition, resulted in four latent classes based on their similarity of the variance.

**Conclusion:**

The mixture variance model provides a realistic and flexible estimate for the variance of gene expression data under limited replicates. We believe that in using the latent class variances, estimated from a larger number of genes in each derived latent group, the *p*-values obtained are more robust than either using a constant gene or gene-specific variance estimate.

## Background

The recent advancement of deoxyribonucleic acid (DNA) microarray technology allows the measurement of expression levels of tens of thousands of genes simultaneously [1,2]. A DNA microarray experiment measures the abundance of messenger ribonucleic acid (mRNA) present in a set of cells, and a high concentration of mRNA for a given gene indicates a high expression level for that gene [3,4]. The solution of mRNA is either radiolabelled or fluorescently labelled and then allowed to hybridize to spots on the array. Further information about early DNA microarray experiments can be found in [5]. There is a wide variety of arrays, but the two main kinds are short and long oligonucleotide arrays. In oligonucleotide arrays, there are approximately 20 probes pairs of a perfect match (PM) and a mismatch (MM) for each gene. The PM probe contains a match to a small subsequence of a gene's polynucleotide, about 25 bases long, while the MM probe acts as a control, being a copy of the PM probe with the central position flipped to its complement. The amount of mRNA present in the target gene in a sample is estimated from a combination (usually, the average) of the PM-MM intensity differences over the 20 probe pairs [6].

In a spotted complimentary DNA (cDNA) or long oligonucleotide arrays (the kind featured in this article) thousands of spots of cDNA from the genes are printed onto a glass slide or some other form of substrate. Two different mRNA samples are separately reverse-transcribed into cDNA and labelled with different fluorescent dyes, green (cyanine 3) or red (cyanine 5). The mixture of labelled cDNA is co-hybridized on the same microarray, and the labelled cDNA molecule will bind to the complementary fragments of cDNA sequence on the slide. A laser scanner is then used to measure both fluorescent signals emitted at each spot on the chip. The general idea assumed behind the technology is that if a particular gene is highly expressed in the sample, it produces many molecules of mRNA [4]. These in turn will hybridize to the probes on the microarray and generate a very bright fluorescent area. Genes that are less expressed produce less mRNA, which results in dimmer fluorescent spots. If there is no fluorescence, no messenger molecules have hybridized to the probes, indicating that the gene is inactive. By comparing the intensity levels of the emitted fluorescent lights between the samples, it is hoped that one might be able to identify any differences in the gene expression profiles of the various samples.

In a typical cDNA microarray experiment, we are looking to ascertain whether gene *i *displays differential expression between two samples *T *and *C*, labelled with differential colours red and green. For instance, these experiments include comparing tumor and normal tissue cells, treated and untreated cells, or cells from *knockout *and *wild-type *organisms. The samples can be compared on the same slide (*i.e*. same hybridization), resulting in a *direct estimate *of differences in expression levels since the measurements come from the same hybridization. An alternative is when expression levels *T*_*i *_and *C*_*i *_are estimated in two different hybridizations, with *T *together with reference sample *R *and *C *with another reference sample *R'*. This is an *indirect estimate *of the gene's differential expression since the *T *and *C *expression levels are from different hybridizations [7]. Sometimes, common reference samples are hybridized with each mRNA sample of interest (*T *or *C*), resulting in what are known as *common reference designs*. The common reference sample could be tissues from *wild-type *organisms or control tissue or a pool of all the samples of interest.

### Standard statistical analyses

The simplest way, used by many in the field, to ascertain a gene's differential expression, is on the basis of a *fold-change *criterion, defined by the log-ratio *δ*_*i *_= log_2_(*T*_*i*_/*C*_*i*_), under direct comparison or *δ*_*i *_= log_2_(*T*_*i*_/*R*_*i*_) - log_2_(*C*_*i*_/R′i
 MathType@MTEF@5@5@+=feaafiart1ev1aaatCvAUfKttLearuWrP9MDH5MBPbIqV92AaeXatLxBI9gBaebbnrfifHhDYfgasaacH8akY=wiFfYdH8Gipec8Eeeu0xXdbba9frFj0=OqFfea0dXdd9vqai=hGuQ8kuc9pgc9s8qqaq=dirpe0xb9q8qiLsFr0=vr0=vr0dc8meaabaqaciaacaGaaeqabaqabeGadaaakeaacuWGsbGugaqbamaaBaaaleaacqWGPbqAaeqaaaaa@2F6C@) for indirect comparison. It is expected that the majority of genes will have a *δ*_*i *_value close to 0 [4]. Those genes with a large positive *δ*_*i *_value (*δ*_*i *_> 1) are generally concluded to be *overexpressed *or *upregulated *in the *T *sample, and those whose *δ*_*i *_is negatively large (*δ*_*i *_< -1) are concluded to be *underexpressed *or *downregulated *in the *T *sample. However, the use of fold change is of limited use, as the intensities are associated with some biological, experimental and measurement error, and unless these error distributions can be derived, it is difficult to assess whether a ratio of 1.9, say, is worth noting or whether it has occurred by chance. Furthermore, the boundaries accepted for thresholding these fold-changes seem to be rather arbitrary and very little documentation can be found to support these criteria.

In recent times, the identification of genes that are differentially expressed between two conditions has been based on a standardised fold-change, which is the fold-change divided by an estimate of its standard deviation. A *t*-test, with a correction for multiple testing, is then used to test for significance of the standardised fold-change. This allows for the assessment of significance of the observed differences in the presence of all the sources of variation, which are not necessarily equal from gene to gene. Our contribution to the problem of identifying genuinely differentially expressed genes is on the estimation of reliable standard deviations of gene expression levels. Modelling of gene expression variability ranges between two extreme cases: a constant variance, which is too stringent an assumption, to independent gene-specific variances. The latter option has low power as it is based on very few replications as a result of the relatively large cost of commercial microarray chips. This makes estimation of the sample standard deviation very unreliable and unstable. An ad-hoc solution to the problem includes discarding genes with a small fold-change and very small standard deviations [4]. A better method, called *Significance Analysis of Microarrays *(SAM), developed in [8], adds a constant *a*_0 _to each gene-specific standard deviation, thus preventing the denominator of the *t*-statistic from getting too small. This was expounded in [3], using an empirical Bayesian procedure, by taking *a*_0 _equal to the 90*th *percentile of the standard deviations of all the genes. Shrinking the gene-specific standard deviations in this way helps to minimize the false discovery rates (i.e. a large *t *statistic).

### Mixture models

An intuitive approach to modelling gene expression data is to assume two groups of genes, one group with genes that are differentially expressed and the other with genes that are not differentially expressed. This approach has been used in analyses involving mixture models for gene expression levels. Formally, the basic assumption is that the distribution of the difference *δ*_*i *_can be flexibly modelled as a mixture with two components: a subgroup of genes with *δ*_*i *_around 0 and a subgroup of genes with non zero *δ*_*i *_[3,4,9,10]. Using this approach, Lonnstedt and Speed [4], derived an empirical Bayesian statistic *B*, which is the log posterior odds of differential expression. Efron et al. [3] and Efron and Tibshirani [10] also consider a two component mixture model to model differential gene expression, the later using a rank-based nonparametric two-sample test statistic. A similar approach was followed-up in [11] based on a fully Bayesian hierarchical model, but with an unknown number of mixture components. The number of components was treated as a random variable and estimated with the other parameters based on the pioneering work of Richardson and Green [12]. Other approaches using mixture models for gene expression data from microarray experiments can be found in [13,14].

### Purpose of the paper

A number of methods, particularly based on full Bayesian hierarchical models, have been used to provide better estimates of variance for gene differential data. These methods provide estimates of gene-specific variance, which are the weighted average of the empirical variance and a prior variance estimate [11,15,16]. In particular, Lewin *et al*. [16] provide a fully Bayesian approach combining estimation of gene differential expression, biological variability and array effects with a hierarchical prior distribution on gene-specific variances. Other than modelling the gene-specific variances with an exchangeable hierarchical prior, Delmar et al. [17] use a mixture model on the distribution of gene-specific variances. Genes are grouped into latent classes based on homogeneity of their variances. A gene is assigned a variance based on its latent class membership and this variance is estimated with high power because of the large number of genes (hence a larger number of replicates) in that latent class. All these methods produce what are called *regularized t*-tests.

Our work is closely related to that proposed by Delmar et al. [17], who used the EM-algorithm to fit a variance mixture model for gene expression data. We believe our Bayesian approach has certain advantages and adds value in comparison to the EM-algorithm approach. In using a Bayesian hierarchical model, we are able to model various sources of variability in a common model, thus propagating uncertainty. Within a Bayesian hierarchical model framework, it is far easier to borrow and share data across all genes in order to obtain more reliable estimates of their variance and at the same time allowing for some variability. In this approach, variances are stabilized and shrunk towards the average variance within each component of the mixture, in particular some small and large variance estimates that are incompatible with the overall distribution are increased and decreased, respectively.

Furthermore, in complex biological data exhibiting a lot of noise, traditional statistical methods, such as the EM-algorithm, can struggle to cope with complex non-linear models when used to explore such data. In the Bayesian paradigm, on the other hand, all the unknown quantities are treated together in a consistent manner, to give fully probabilistic information on all unobserved variables, even their functions. Our method is based on a Chi-squared (*χ*^2^)-mixture model for the gene-specific variances, with the number of components ranging from 1 to 5. In bioinformatics, as in many fields, mixture models have been fitted through the expectation-maximisation (EM) algorithm with different values of the number of mixture components [9,17,18]. In this paper, we present a Bayesian analysis of the variance mixture model, which we implement in the Bayesian software package, WinBUGS [19]. The methodology is applied to a dataset on diffuse large B-cell lymphomas. The data contains expression levels of chronic lymphatic leukaemia (CLL) and diffuse large cell (DLCL) malignancies for the 9216 genes under study. The results of applying our model to the data-set are presented in the *Results from the mixture model *section. A discussion of the model and the results are in the *Conclusions *section.

It is hoped that the work presented in this paper will contribute to a large volume of current research work aimed at minimizing the risk of false positives in microarray experiments. The two extreme assumptions on the variance of gene expression data are presented in the *Standard models for gene variance *section and a description of the variance mixture model is contained in *A mixture model for gene variance *section.

## Results and discussion

### Description of the data set

We analyse the data described in [20], which investigates the classification of diffuse large B-cell lymphomas into distinct groups by gene expression profiling. Diffuse large B-cell lymphoma is an aggressive malignancy of mature B lymphocytes. It is estimated that, with an annual incidence of over 25,000 cases, it accounts for approximately 40% of all cases of non-Hodgkin's lymphoma. Currently, a combination of clinical parameters is used to provide an assessment of a patient's risk profile and to determine the most suitable clinical course of treatment. Whilst most patients initially respond well to chemotherapy, patients receiving the same diagnosis can have very different final outcomes in terms of remission achieved and their overall survival. It is suspected that the prognostic variables used are in fact proxies for the underlying cellular and molecular variation within diffuse large B-cell lymphomas. Their work considers whether gene expression profiling could subdivide this 'clinically heterogenous diagnostic category into molecularly distinct diseases that would possess more homogeneous clinical behaviours' [20]. The microarrays used in this experiment were specially designed complementary DNA microarrays, called 'Lymphochips', which included those genes with a known or suspected role in processes that are important in immunology or cancer, and those genes known to be preferentially expressed in lymphoid cells. The profiling of gene expression included the three most prevalent adult lymphoid malignancies, in addition to profiling the gene expression in purified normal lymphocyte subpopulations under a range of activation conditions, in normal human tonsil and lymph node, and in a variety of lymphoma and leukaemia cell lines. From each experimental mRNA sample, a cDNA sample was prepared and labelled with red Cy5 dye. Furthermore, a corresponding reference cDNA sample, labelled with green Cy3 dye, was prepared from a pool of mRNAs isolated from nine different lymphoma cell lines. The labelled samples were combined and hybridized to the microarray.

Approximately 1.8-million measurements of gene expression were taken in all, across 96 normal and malignant lymphocyte samples, using 128 of the Lymphochip microarrays. To demonstrate the method proposed in this paper, we use a small subset, containing only eight slides from two conditions. Four slides quantify gene expression relating to chronic lymphatic leukaemia (CLL) malignancies and the other four to diffuse large cell lymphoma (DLCL) malignancies. Each slide contains measurements for 9216 genes. The red-to-green intensity ratio can be quantified for each gene and this reflects the relative abundance of mRNA in the experimental sample compared with the reference mRNA pool. By using a common reference sample, these fluorescent ratios can be considered a comparable measurement of the relative expression level of each gene across all of the samples. We want to compare different gene expression levels between the CLL and DLCL malignancy conditions.

### Results from the mixture model

We fitted the model:

yicrc=μic+εicrc
 MathType@MTEF@5@5@+=feaafiart1ev1aaatCvAUfKttLearuWrP9MDH5MBPbIqV92AaeXatLxBI9gBaebbnrfifHhDYfgasaacH8akY=wiFfYdH8Gipec8Eeeu0xXdbba9frFj0=OqFfea0dXdd9vqai=hGuQ8kuc9pgc9s8qqaq=dirpe0xb9q8qiLsFr0=vr0=vr0dc8meaabaqaciaacaGaaeqabaqabeGadaaakeaacqWG5bqEdaWgaaWcbaGaemyAaKMaem4yamMaemOCai3aaSbaaWqaaiabdogaJbqabaaaleqaaOGaeyypa0dcciGae8hVd02aaSbaaSqaaiabdMgaPjabdogaJbqabaGccqGHRaWkcqWF1oqzdaWgaaWcbaGaemyAaKMaem4yamMaemOCai3aaSbaaWqaaiabdogaJbqabaaaleqaaaaa@41EC@

where yicrc
 MathType@MTEF@5@5@+=feaafiart1ev1aaatCvAUfKttLearuWrP9MDH5MBPbIqV92AaeXatLxBI9gBaebbnrfifHhDYfgasaacH8akY=wiFfYdH8Gipec8Eeeu0xXdbba9frFj0=OqFfea0dXdd9vqai=hGuQ8kuc9pgc9s8qqaq=dirpe0xb9q8qiLsFr0=vr0=vr0dc8meaabaqaciaacaGaaeqabaqabeGadaaakeaacqWG5bqEdaWgaaWcbaGaemyAaKMaem4yamMaemOCai3aaSbaaWqaaiabdogaJbqabaaaleqaaaaa@33F1@ is gene *i*'s log-ratio of observed intensity for condition *c *to the reference mRNA pool in replicate *r*_*c*_(*i *= 1, ..., 9216; *c *= 1 for CLL, 2 for DLCL; *r*_1 _= 1, ..., 4; *r*_2 _= 1, ..., 4);  μ_ic_ is the mean log-ratio; and εicrc
 MathType@MTEF@5@5@+=feaafiart1ev1aaatCvAUfKttLearuWrP9MDH5MBPbIqV92AaeXatLxBI9gBaebbnrfifHhDYfgasaacH8akY=wiFfYdH8Gipec8Eeeu0xXdbba9frFj0=OqFfea0dXdd9vqai=hGuQ8kuc9pgc9s8qqaq=dirpe0xb9q8qiLsFr0=vr0=vr0dc8meaabaqaciaacaGaaeqabaqabeGadaaakeaaiiGacqWF1oqzdaWgaaWcbaGaemyAaKMaem4yamMaemOCai3aaSbaaWqaaiabdogaJbqabaaaleqaaaaa@3424@ is normally distributed with mean 0.

The mixture weights were estimated as part of the model, where they were assigned a Dirichlet prior distribution, (*π*_1_, ..., *π*_*k*_) ~ Dirichlet(1, ..., 1), where *k *is the number of mixture components. The *χ*^2 ^mixture scale parameters *ψ*_*j *_were assigned independent Gamma(0.01, 0.01) prior distributions. For each mixture model, three independent chains were run for 50,000 iterations. We discarded the first 20,000 iterations and used a combined sample of the remaining 60,000 iterations for posterior summaries.

The determination of the number of components to include in a mixture is an important, yet unresolved key issue in finite mixture models. There are a number of approaches for assessing the adequate number of mixture components, and a review of the available approaches is given in Oliveira-Brochado and Martins [21]. Standard *χ*^2 ^based goodness of fit and likelihood ratio tests are not reliable statistics for deciding the number of mixture components; in particular the likelihood has a tendency to select more complex models, having a higher number of parameters. The widely used selection statistics – Bayesian Information Criterion (BIC) and Akaike Information Criterion (AIC) – impose a penalty on the likelihood to account for the number of parameters estimated in the model. Both BIC and AIC have been shown to be suitable in determining the number of latent classes in the normal-mixture model. However, in some non-normal mixture models, such as beta-mixture, BIC and AIC were shown to perform poorly, by selecting excessive numbers of components whilst an alternative criterion, the Integrated Completed Likelihood (ICL)-BIC selected the right number of mixture components in simulation studies [18]. On the other hand, the ICL-BIC selected too few components in Poisson-mixture models [22], whereby the performance of BIC and AIC criteria were adequate, more so for the BIC in large samples. For the purpose of this Bayesian application, and considering that the underlying distribution of variance is continuous and sample size is relatively large, we chose to use the BIC criterion for model selection. The BIC is defined as: BIC = -2 * log -likelihood + *P *log *g*, where *P *is the number of unknown parameters in the model (*P *= 2 * *k *- 1 in our case) and *g *is the sample size. In the Bayesian context, BIC selects a model that is most probable after conditioning on the data [22]. The model with the smallest BIC value is selected to be the model that best predicts the number of mixture components, bearing in mind that when a set of models estimated on the same data set yield the same log -likelihood value, BIC favours parsimony by selecting the model with the fewer parameters. We present posterior mean and standard deviation (SD) estimates of the model parameters from fitting the proposed model with corresponding values of BIC (see Table 1). The results are shown only up to the four-component mixture model as the BIC value changed by only about 2 when fitting a five-component mixture model. Also provided are comparative results from fitting the EM algorithm [17]. Our Bayesian mixture variance model gives very similar results to those obtained from the EM algorithm. The results seem to imply that there is one small latent class consisting of genes with a large variance.

**Table 1:** Posterior mean (SD) for various variance mixture models

Model	Bayesian Model		EM algorithm	
	π_*j*_	σ¯ MathType@MTEF@5@5@+=feaafiart1ev1aaatCvAUfKttLearuWrP9MDH5MBPbIqV92AaeXatLxBI9gBaebbnrfifHhDYfgasaacH8akY=wiFfYdH8Gipec8Eeeu0xXdbba9frFj0=OqFfea0dXdd9vqai=hGuQ8kuc9pgc9s8qqaq=dirpe0xb9q8qiLsFr0=vr0=vr0dc8meaabaqaciaacaGaaeqabaqabeGadaaakeaaiiGacuWFdpWCgaqeaaaa@2E8E@_*j*_	π_*j*_	σ¯ MathType@MTEF@5@5@+=feaafiart1ev1aaatCvAUfKttLearuWrP9MDH5MBPbIqV92AaeXatLxBI9gBaebbnrfifHhDYfgasaacH8akY=wiFfYdH8Gipec8Eeeu0xXdbba9frFj0=OqFfea0dXdd9vqai=hGuQ8kuc9pgc9s8qqaq=dirpe0xb9q8qiLsFr0=vr0=vr0dc8meaabaqaciaacaGaaeqabaqabeGadaaakeaaiiGacuWFdpWCgaqeaaaa@2E8E@_*j*_
*2 Classes*				
1	0.2276 (0.009)	0.7624 (0.008)	0.2286	0.7607
2	0.7724 (0.0085)	0.3426 (0.0024)	0.7713	0.3422
*BIC*	22911.89		22657.89	
*3 Classes*				
*1*	0.0677 (0.0039)	1.0220 (0.2627)	0.0688	1.0225
2	0.4462 (0.0170)	0.4977 (0.0077)	0.4546	0.4970
3	0.4860 (0.0182)	0.2841 (0.0035)	0.4766	0.2839
*BIC*	21819.82		21703.82	
*4 Classes*				
1	0.2303 (0.0146)	0.6125 (0.0129)	0.2317	0.6125
2	0.0384 (0.0045)	1.1547 (0.0321)	0.0370	1.1523
3	0.2318 (0.0218)	0.2413 (0.0053)	0.2285	0.2427
4	0.4994 (0.0201)	0.3775 (0.0161)	0.5028	0.3782
*BIC*	21727.75		21620.86	

For each fitted variance mixture model, we assigned each gene *i *to group *j *according to the highest estimated posterior classification probability π_*ij*_. In turn, gene *i *was assigned variance σ^i∗2=σ^j2
 MathType@MTEF@5@5@+=feaafiart1ev1aaatCvAUfKttLearuWrP9MDH5MBPbIqV92AaeXatLxBI9gBaebbnrfifHhDYfgasaacH8akY=wiFfYdH8Gipec8Eeeu0xXdbba9frFj0=OqFfea0dXdd9vqai=hGuQ8kuc9pgc9s8qqaq=dirpe0xb9q8qiLsFr0=vr0=vr0dc8meaabaqaciaacaGaaeqabaqabeGadaaakeaaiiGacuWFdpWCgaqcamaaDaaaleaacqWGPbqAcqGHxiIkaeaacqaIYaGmaaGccqGH9aqpcuWFdpWCgaqcamaaDaaaleaacqWGQbGAaeaacqaIYaGmaaaaaa@3749@ where σ^j2
 MathType@MTEF@5@5@+=feaafiart1ev1aaatCvAUfKttLearuWrP9MDH5MBPbIqV92AaeXatLxBI9gBaebbnrfifHhDYfgasaacH8akY=wiFfYdH8Gipec8Eeeu0xXdbba9frFj0=OqFfea0dXdd9vqai=hGuQ8kuc9pgc9s8qqaq=dirpe0xb9q8qiLsFr0=vr0=vr0dc8meaabaqaciaacaGaaeqabaqabeGadaaakeaaiiGacuWFdpWCgaqcamaaDaaaleaacqWGQbGAaeaacqaIYaGmaaaaaa@3102@ is the estimated variance of latent class *j*. Thus, the derived *t*-statistic for gene *i *now becomes:

$$t_{i}^{*}={\bar{\delta }}_{i}/SE({\bar{\delta }}_{i})={\bar{\delta }}_{i}/{\left({\hat{\sigma }}_{i*}^{2}/m_{1}+{\hat{\sigma }}_{i*}^{2}/m_{2}\right)}^{1/2}.$$

where _m1_ and _m2_ are sample sizes  for condition 1 and 2, respectively.

**Figure 1:**
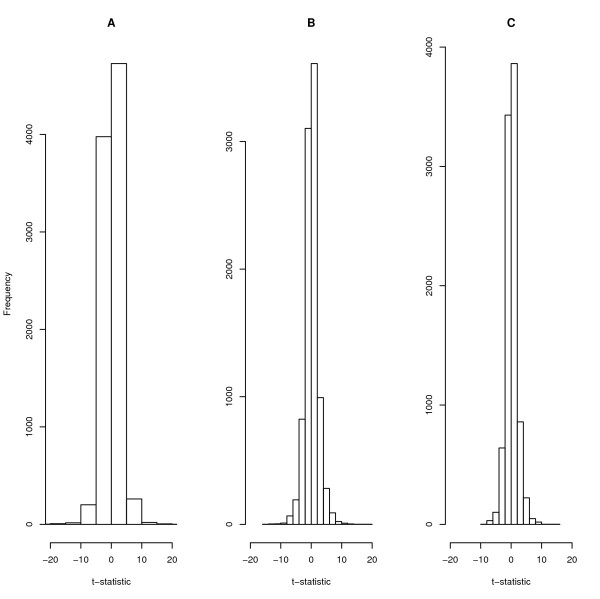
**Distribution of the *t*-statistic, using different estimate of gene standard error**. Plot (A) is a histogram based on gene-specific standard error, (B) is based on the four-component mixture model estimated standard error and (C) shows a histogram based on homogeneous standard error.

We compared the *t*-statistics using gene-specific and homogeneous standard errors to the four-component mixture estimated standard errors. It was evident that the majority of genes have *t*-statistics very close to 0, indicating similar expression levels under the two conditions, CLL and DLCL (see Figure 1). All three have a similar shape; however, the mixture estimated *t*-statistic is more conservative than the gene-specific *t*-statistics (with its own problem of not having enough data to estimate the variance) and not as naive as the homogeneous statistic, which is based on overly unrealistic gene variance assumptions. The mixture estimated *t*-statistic is more informative as it uses standard errors estimated from a larger number of genes based on their variance similarity. In the data set, some genes have larger variances than others and large variance genes that are not differentially expressed are more likely to have large log fold changes. However, when taking variance into account, these genes produce small *t*-statistics. On the other hand, with only four measurements per group, the estimate of the standard error is not stable and some genes have large *t*-statistics only because, by chance, the denominator was very small. This relative large disparity in *t*-statistics is demonstrated in histogram (A) and (C). The histogram (B)is an improvement in that it does give high *t*-statistics to genes only because they have small sample variances. The statistic presented in histogram (B) is referred to as the *modified t*-statistic, which are based on borrowed strength across genes in order to obtain more stable estimates of gene variances.

This classification of genes under the Bayesian mixture model was compared to that obtained under the EM mixture model for the four component model (see Table 2). There is very good agreement between the two models, with only 387 out of 9216 genes classified differently. The measure of agreement, *kappa*, was 0.929. For all of the 387 genes that were classified differently, their two most likely classes, based on the posterior classification π_*ij*_, were the same in both models. In this case, one can conclude that the two models resulted in agreement in classifying genes to the four mixture components.

**Table 2:** Classification of genes under the Bayesian and EM mixture models, with four components

EM model	Bayesian model				Total
	1	2	3	4	
1	1621	3	0	52	1676
2	18	242	0	0	260
3	0	0	1848	161	2009
4	42	0	111	5118	5271
Total	1681	245	1959	5331	9216

We identified the top ten ranked genes according to the absolute *t*-statistic obtained from using various variance models (see Table 3). Only two genes, coded 2143 and 4323 are consistently ranked in the top ten across all variance models. Genes coded 3181, 4069, 4532, 4586 and 8076 are ranked in the top ten in at least two variance models.

**Table 3:** Top ten ranked genes by different variance models (Genes are listed by their codes)

Gene-specific	Constant	2 Classes	3 Classes	4 Classes	Weighted
4323	2602	1939	2143	2143	2143
4532	1945	2143	3181	4069	4069
4069	257	3181	4069	4323	4323
8076	1400	7161	8903	4532	4532
4331	1939	2003	4323	7496	8076
6635	2143	4069	4347	8076	3181
2026	3181	6731	4532	4586	4586
2143	7003	6343	6592	5674	8903
4586	4323	8649	8628	8048	2542
8892	3151	4323	5746	1631	5674

Finally, other than setting σ^i∗2=σ^j2
 MathType@MTEF@5@5@+=feaafiart1ev1aaatCvAUfKttLearuWrP9MDH5MBPbIqV92AaeXatLxBI9gBaebbnrfifHhDYfgasaacH8akY=wiFfYdH8Gipec8Eeeu0xXdbba9frFj0=OqFfea0dXdd9vqai=hGuQ8kuc9pgc9s8qqaq=dirpe0xb9q8qiLsFr0=vr0=vr0dc8meaabaqaciaacaGaaeqabaqabeGadaaakeaaiiGacuWFdpWCgaqcamaaDaaaleaacqWGPbqAcqGHxiIkaeaacqaIYaGmaaGccqGH9aqpcuWFdpWCgaqcamaaDaaaleaacqWGQbGAaeaacqaIYaGmaaaaaa@3749@, which is outright membership, we also considered a weighted variance estimate σ^iw2=∑πijσj2
 MathType@MTEF@5@5@+=feaafiart1ev1aaatCvAUfKttLearuWrP9MDH5MBPbIqV92AaeXatLxBI9gBaebbnrfifHhDYfgasaacH8akY=wiFfYdH8Gipec8Eeeu0xXdbba9frFj0=OqFfea0dXdd9vqai=hGuQ8kuc9pgc9s8qqaq=dirpe0xb9q8qiLsFr0=vr0=vr0dc8meaabaqaciaacaGaaeqabaqabeGadaaakeaaiiGacuWFdpWCgaqcamaaDaaaleaacqWGPbqAcqWG3bWDaeaacqaIYaGmaaGccqGH9aqpdaaeabqaaiab=b8aWnaaBaaaleaacqWGPbqAcqWGQbGAaeqaaOGae83Wdm3aa0baaSqaaiabdQgaQbqaaiabikdaYaaaaeqabeqdcqGHris5aaaa@3E70@ based on a four-component variance mixture model. We ranked genes using this weighted variance estimate to compare the ranking obtained from the other variance estimates (see Table 3). Eight genes coded 2143, 3181, 4069, 4323, 4532, 4586, 8076 and 8903 that appear in the top ten under the weighted variance model, also appear at least once in the top ten of the other variance models. These genes might be interesting, thus requiring further analysis and investigation.

We are aware that different assumptions for the prior distributions for both mixture weights and scales may give different results. We performed some limited sensitivity examination of the results to different specification of the priors. There were slight differences in the results, but the substantive conclusions were not affected.

## Conclusion

We have presented a Bayesian variance mixture model for differential gene expression data. This model is a compromise between two extreme models: the too stringent constant gene variance and the overparameterised gene-specific variance models, which are both unrealistic assumptions. Our mixture variance model provides a more realistic and flexible estimate for the variance of gene expression data under limited replicates. We believe that in using the (weighted) latent class variances, estimated from a larger number of genes in each derived latent group, the *p*-values obtained are more accurate then either using a constant gene variance or gene-specific variance estimate.

In our example data, the results obtained from using our model are in close agreement to those obtained using EM algorithm implementation [17], which had been shown, using simulation studies, to be flexible and reliable in both true and false discovery rates. Our results are based on the assumptions of normally distributed log-ratios and a constant gene-variance between the two conditions. We are working on relaxing these conditions, in particular in using a long-tailed *t*-distribution as a robust alternative to allow for the possibility of gene-intensity measurement outliers. By varying the degrees of freedom, the *t*-distribution can also be used to investigate the sensitivity of the posterior results to changes in the prior for the gene intensity measurements.

## Methods

### Notation and variance models

We assume that the intensity level data is background corrected and normalised according to [23], using an arcsinh transformation based on a model for the dependence of the variance on the mean intensity levels with variance stabalizing properties. These are implemented in a variance stabilizing transformation vsn, the title function of the vsn package, part of the Bioconductor project for R [24]. This results in generalised log-transformed expression intensity values. We develop the methodology for unpaired (*indirect*) data case, where two samples of interest are each co-hybridised with a reference sample. That is, each independent slide is a two-colour microarray experiment. The methodology is easily adapted to paired (*direct comparison*) data case.

We consider a pair of log-transformed expression measurements (*T*_*ir*_, *R*_*ir*_) for gene *i *: *i *= 1, ..., *g *in replicate *r *: *r *= 1, ..., *m*_*T *_for condition *T *co-hybridized with reference sample *R*. Then, *y*_*itr *_= *T*_*ir *_- *R*_*ir *_is the log-ratio of gene *i *under condition *T *in replicate *r*. Similarly, let (*C*_*ir'*_, R′ir′
 MathType@MTEF@5@5@+=feaafiart1ev1aaatCvAUfKttLearuWrP9MDH5MBPbIqV92AaeXatLxBI9gBaebbnrfifHhDYfgasaacH8akY=wiFfYdH8Gipec8Eeeu0xXdbba9frFj0=OqFfea0dXdd9vqai=hGuQ8kuc9pgc9s8qqaq=dirpe0xb9q8qiLsFr0=vr0=vr0dc8meaabaqaciaacaGaaeqabaqabeGadaaakeaacuWGsbGugaqbamaaBaaaleaacqWGPbqAcuWGYbGCgaqbaaqabaaaaa@30E5@) be a pair of expression measurements for gene *i *in replicate *r' *: *r' *= 1, ..., *m*_*C *_for condition *C *co-hybridized with reference sample *R'*. The difference *y*_*icr' *_= *C*_*ir' *_- R′ir′
 MathType@MTEF@5@5@+=feaafiart1ev1aaatCvAUfKttLearuWrP9MDH5MBPbIqV92AaeXatLxBI9gBaebbnrfifHhDYfgasaacH8akY=wiFfYdH8Gipec8Eeeu0xXdbba9frFj0=OqFfea0dXdd9vqai=hGuQ8kuc9pgc9s8qqaq=dirpe0xb9q8qiLsFr0=vr0=vr0dc8meaabaqaciaacaGaaeqabaqabeGadaaakeaacuWGsbGugaqbamaaBaaaleaacqWGPbqAcuWGYbGCgaqbaaqabaaaaa@30E5@ is the log-ratio for gene *i *under condition *C*. In such an experiment, we want to compare the log-ratios *y*_*itr *_and *y*_*icr' *_between the *T *and *C *samples. We can obtain sample data statistics for each condition, such as averages:

T¯i=1mT∑r=1mTyitrC¯i=1mC∑r′=1mCyicr′
 MathType@MTEF@5@5@+=feaafiart1ev1aaatCvAUfKttLearuWrP9MDH5MBPbIqV92AaeXatLxBI9gBaebbnrfifHhDYfgasaacH8akY=wiFfYdH8Gipec8Eeeu0xXdbba9frFj0=OqFfea0dXdd9vqai=hGuQ8kuc9pgc9s8qqaq=dirpe0xb9q8qiLsFr0=vr0=vr0dc8meaabaqaciaacaGaaeqabaqabeGadaaakeaafaqadeGabaaabaGafmivaqLbaebadaWgaaWcbaGaemyAaKgabeaakiabg2da9maalaaabaGaeGymaedabaGaemyBa02aaSbaaSqaaiabdsfaubqabaaaaOWaaabCaeaacqWG5bqEdaWgaaWcbaGaemyAaKMaemiDaqNaemOCaihabeaaaeaacqWGYbGCcqGH9aqpcqaIXaqmaeaacqWGTbqBdaWgaaadbaGaemivaqfabeaaa0GaeyyeIuoaaOqaaiqbdoeadzaaraWaaSbaaSqaaiabdMgaPbqabaGccqGH9aqpdaWcaaqaaiabigdaXaqaaiabd2gaTnaaBaaaleaacqWGdbWqaeqaaaaakmaaqahabaGaemyEaK3aaSbaaSqaaiabdMgaPjabdogaJjqbdkhaYzaafaaabeaaaeaacuWGYbGCgaqbaiabg2da9iabigdaXaqaaiabd2gaTnaaBaaameaacqWGdbWqaeqaaaqdcqGHris5aaaaaaa@581F@

and variances:

SiT2=1mT−1∑r=1mT(yitr−T¯i)2SiC2=1mC−1∑r′=1mC(yicr′−C¯i)2.
 MathType@MTEF@5@5@+=feaafiart1ev1aaatCvAUfKttLearuWrP9MDH5MBPbIqV92AaeXatLxBI9gBaebbnrfifHhDYfgasaacH8akY=wiFfYdH8Gipec8Eeeu0xXdbba9frFj0=OqFfea0dXdd9vqai=hGuQ8kuc9pgc9s8qqaq=dirpe0xb9q8qiLsFr0=vr0=vr0dc8meaabaqaciaacaGaaeqabaqabeGadaaakeaafaqadeGabaaabaGaem4uam1aa0baaSqaaiabdMgaPjabdsfaubqaaiabikdaYaaakiabg2da9maalaaabaGaeGymaedabaGaemyBa02aaSbaaSqaaiabdsfaubqabaGccqGHsislcqaIXaqmaaWaaabCaeaacqGGOaakcqWG5bqEdaWgaaWcbaGaemyAaKMaemiDaqNaemOCaihabeaakiabgkHiTiqbdsfauzaaraWaaSbaaSqaaiabdMgaPbqabaGccqGGPaqkdaahaaWcbeqaaiabikdaYaaaaeaacqWGYbGCcqGH9aqpcqaIXaqmaeaacqWGTbqBdaWgaaadbaGaemivaqfabeaaa0GaeyyeIuoaaOqaaiabdofatnaaDaaaleaacqWGPbqAcqWGdbWqaeaacqaIYaGmaaGccqGH9aqpdaWcaaqaaiabigdaXaqaaiabd2gaTnaaBaaaleaacqWGdbWqaeqaaOGaeyOeI0IaeGymaedaamaaqahabaGaeiikaGIaemyEaK3aaSbaaSqaaiabdMgaPjabdogaJjqbdkhaYzaafaaabeaakiabgkHiTiqbdoeadzaaraWaaSbaaSqaaiabdMgaPbqabaGccqGGPaqkdaahaaWcbeqaaiabikdaYaaaaeaacuWGYbGCgaqbaiabg2da9iabigdaXaqaaiabd2gaTnaaBaaameaacqWGdbWqaeqaaaqdcqGHris5aOGaeiOla4caaaaa@6DFD@

The selection of differentially expressed genes can proceed simply by a test based on *log fold-change criterion*, δ¯i=T¯i−C¯i
 MathType@MTEF@5@5@+=feaafiart1ev1aaatCvAUfKttLearuWrP9MDH5MBPbIqV92AaeXatLxBI9gBaebbnrfifHhDYfgasaacH8akY=wiFfYdH8Gipec8Eeeu0xXdbba9frFj0=OqFfea0dXdd9vqai=hGuQ8kuc9pgc9s8qqaq=dirpe0xb9q8qiLsFr0=vr0=vr0dc8meaabaqaciaacaGaaeqabaqabeGadaaakeaaiiGacuWF0oazgaqeamaaBaaaleaacqWGPbqAaeqaaOGaeyypa0JafmivaqLbaebadaWgaaWcbaGaemyAaKgabeaakiabgkHiTiqbdoeadzaaraWaaSbaaSqaaiabdMgaPbqabaaaaa@377C@. However, as pointed out in the *Standard statistical analyses *section, the use of fold changes is limited because the intensities are associated with biological, experimental and measurement errors. Formally, we consider significance testing by assuming that the observed log-ratios *y*_*itr *_and *y*_*itr' *_are modelled by simple linear models:

*y*_*itr *_= *μ*_*iT *_+ *ε*_*itr *_and *y*_*icr' *_= *μ*_*iC *_+ *ε*_*icr' *_

where *ε*_*itr *_and *ε*_*icr' *_are normally distributed with mean 0 and equal variance σi2
 MathType@MTEF@5@5@+=feaafiart1ev1aaatCvAUfKttLearuWrP9MDH5MBPbIqV92AaeXatLxBI9gBaebbnrfifHhDYfgasaacH8akY=wiFfYdH8Gipec8Eeeu0xXdbba9frFj0=OqFfea0dXdd9vqai=hGuQ8kuc9pgc9s8qqaq=dirpe0xb9q8qiLsFr0=vr0=vr0dc8meaabaqaciaacaGaaeqabaqabeGadaaakeaaiiGacqWFdpWCdaqhaaWcbaGaemyAaKgabaGaeGOmaidaaaaa@30F0@. For each gene *i*, we use the usual two-sample *t*-test *H*_0 _: *μ*_*iT *_= *μ*_*iC *_against *H*_1 _: *μ*_*iT *_≠ *μ*_*iC*_. This resulting test statistic is based on the standardised fold change:

ti=δ¯i/SE(δ¯i)=δ¯i/SiT2/mT+SiC2/mC)1/2
 MathType@MTEF@5@5@+=feaafiart1ev1aaatCvAUfKttLearuWrP9MDH5MBPbIqV92AaeXatLxBI9gBaebbnrfifHhDYfgasaacH8akY=wiFfYdH8Gipec8Eeeu0xXdbba9frFj0=OqFfea0dXdd9vqai=hGuQ8kuc9pgc9s8qqaq=dirpe0xb9q8qiLsFr0=vr0=vr0dc8meaabaqaciaacaGaaeqabaqabeGadaaakeaacqWG0baDdaWgaaWcbaGaemyAaKgabeaakiabg2da9GGaciqb=r7aKzaaraWaaSbaaSqaaiabdMgaPbqabaGccqGGVaWlcqWGtbWucqWGfbqrcqGGOaakcuWF0oazgaqeamaaBaaaleaacqWGPbqAaeqaaOGaeiykaKIaeyypa0Jaf8hTdqMbaebadaWgaaWcbaGaemyAaKgabeaakiabc+caViabdofatnaaDaaaleaacqWGPbqAcqWGubavaeaacqaIYaGmaaGccqGGVaWlcqWGTbqBdaWgaaWcbaGaemivaqfabeaakiabgUcaRiabdofatnaaDaaaleaacqWGPbqAcqWGdbWqaeaacqaIYaGmaaGccqGGVaWlcqWGTbqBdaWgaaWcbaGaem4qameabeaakiabcMcaPmaaCaaaleqabaGaeGymaeJaei4la8IaeGOmaidaaaaa@56F6@

whose usefulness depends on an accurate estimate of the standard error (*SE*) of δ¯
 MathType@MTEF@5@5@+=feaafiart1ev1aaatCvAUfKttLearuWrP9MDH5MBPbIqV92AaeXatLxBI9gBaebbnrfifHhDYfgasaacH8akY=wiFfYdH8Gipec8Eeeu0xXdbba9frFj0=OqFfea0dXdd9vqai=hGuQ8kuc9pgc9s8qqaq=dirpe0xb9q8qiLsFr0=vr0=vr0dc8meaabaqaciaacaGaaeqabaqabeGadaaakeaaiiGacuWF0oazgaqeaaaa@2E70@_*i*_. Generally, there will be very few replications; thus the estimation of SE would be very unreliable and unstable. One solution is to discard genes with a small fold-change and very small standard deviations to avoid getting overoptimistic significant results.

### Standard models for gene variance

There are two extreme cases to model this variance:

• *Independent gene-specific variance*: In this scenario, σiT2=σiC2=σi2
 MathType@MTEF@5@5@+=feaafiart1ev1aaatCvAUfKttLearuWrP9MDH5MBPbIqV92AaeXatLxBI9gBaebbnrfifHhDYfgasaacH8akY=wiFfYdH8Gipec8Eeeu0xXdbba9frFj0=OqFfea0dXdd9vqai=hGuQ8kuc9pgc9s8qqaq=dirpe0xb9q8qiLsFr0=vr0=vr0dc8meaabaqaciaacaGaaeqabaqabeGadaaakeaaiiGacqWFdpWCdaqhaaWcbaGaemyAaKMaemivaqfabaGaeGOmaidaaOGaeyypa0Jae83Wdm3aa0baaSqaaiabdMgaPjabdoeadbqaaiabikdaYaaakiabg2da9iab=n8aZnaaDaaaleaacqWGPbqAaeaacqaIYaGmaaaaaa@3DC0@ is estimated by:

Si2=((MT−1)SiT2+(mC−1)SiC2))/mT+mC−2)
 MathType@MTEF@5@5@+=feaafiart1ev1aaatCvAUfKttLearuWrP9MDH5MBPbIqV92AaeXatLxBI9gBaebbnrfifHhDYfgasaacH8akY=wiFfYdH8Gipec8Eeeu0xXdbba9frFj0=OqFfea0dXdd9vqai=hGuQ8kuc9pgc9s8qqaq=dirpe0xb9q8qiLsFr0=vr0=vr0dc8meaabaqaciaacaGaaeqabaqabeGadaaakeaacqWGtbWudaqhaaWcbaGaemyAaKgabaGaeGOmaidaaOGaeyypa0JaeiikaGIaeiikaGIaemyta00aaSbaaSqaaiabdsfaubqabaGccqGHsislcqaIXaqmcqGGPaqkcqWGtbWudaqhaaWcbaGaemyAaKMaemivaqfabaGaeGOmaidaaOGaey4kaSIaeiikaGIaemyBa02aaSbaaSqaaiabdoeadbqabaGccqGHsislcqaIXaqmcqGGPaqkcqWGtbWudaqhaaWcbaGaemyAaKMaem4qameabaGaeGOmaidaaOGaeiykaKIaeiykaKIaei4la8IaemyBa02aaSbaaSqaaiabdsfaubqabaGccqGHRaWkcqWGTbqBdaWgaaWcbaGaem4qameabeaakiabgkHiTiabikdaYiabcMcaPaaa@54BC@

which has *ν *= *m*_*T *_+ *m*_*C *_- 2 degrees of freedom. Under the null hypothesis, *μ*_*iT *_= *μ*_*iC*_, the standardised fold-change *t*_*i *_is distributed according to a *t*-distribution with *ν *degrees of freedom. This option has low power as it is based on very few replications. This makes estimation of the sample variances very unreliable and unstable, and this results in less powerful *t*-tests.

• *A constant variance*: In this homoscedastic model, all genes are assumed to have the same variance *σ*^2^, which is estimated by:

S2=1g∑i=1gSi2.
 MathType@MTEF@5@5@+=feaafiart1ev1aaatCvAUfKttLearuWrP9MDH5MBPbIqV92AaeXatLxBI9gBaebbnrfifHhDYfgasaacH8akY=wiFfYdH8Gipec8Eeeu0xXdbba9frFj0=OqFfea0dXdd9vqai=hGuQ8kuc9pgc9s8qqaq=dirpe0xb9q8qiLsFr0=vr0=vr0dc8meaabaqaciaacaGaaeqabaqabeGadaaakeaacqWGtbWudaahaaWcbeqaaiabikdaYaaakiabg2da9maalaaabaGaeGymaedabaGaem4zaCgaamaaqahabaGaem4uam1aa0baaSqaaiabdMgaPbqaaiabikdaYaaaaeaacqWGPbqAcqGH9aqpcqaIXaqmaeaacqWGNbWza0GaeyyeIuoakiabc6caUaaa@3DD7@

This has a large number of replicates, totalling (*m*_*T *_+ *m*_*C*_)*g*. The degrees of freedom of the variance estimate are now *ν *= (*m*_*T *_+ *m*_*C *_- 2)*g*, which makes the statistic *t*_*i *_behave like a standard Normal (0,1) variate. However, a constant variance over all the genes is too unrealistic an assumption, and it increases the risk of a false positive result for a gene with a larger variance. On the other hand, there is a high risk of missing out on a truly differentiated gene having a small variance, and a large differential effect.

### A mixture model for gene variance

Assuming that the log -ratios *y*_*itr *_and *y*_*icr' *_follow a normal distribution, then under the null hypothesis *μ*_*iT *_= *μ*_*iC *_the observed scaled gene-specific residual sum of squares *w*_*i *_= νSi2
 MathType@MTEF@5@5@+=feaafiart1ev1aaatCvAUfKttLearuWrP9MDH5MBPbIqV92AaeXatLxBI9gBaebbnrfifHhDYfgasaacH8akY=wiFfYdH8Gipec8Eeeu0xXdbba9frFj0=OqFfea0dXdd9vqai=hGuQ8kuc9pgc9s8qqaq=dirpe0xb9q8qiLsFr0=vr0=vr0dc8meaabaqaciaacaGaaeqabaqabeGadaaakeaaiiGacqWF9oGBcqWGtbWudaqhaaWcbaGaemyAaKgabaGaeGOmaidaaaaa@3214@ is a scaled *Chi*-squared (*χ*^2^) variate with *ν *degrees of freedom, denoted by wi~ψi−1χν2
 MathType@MTEF@5@5@+=feaafiart1ev1aaatCvAUfKttLearuWrP9MDH5MBPbIqV92AaeXatLxBI9gBaebbnrfifHhDYfgasaacH8akY=wiFfYdH8Gipec8Eeeu0xXdbba9frFj0=OqFfea0dXdd9vqai=hGuQ8kuc9pgc9s8qqaq=dirpe0xb9q8qiLsFr0=vr0=vr0dc8meaabaqaciaacaGaaeqabaqabeGadaaakeaacqWG3bWDdaWgaaWcbaGaemyAaKgabeaakiabc6ha+HGaciab=H8a5naaDaaaleaacqWGPbqAaeaacqGHsislcqaIXaqmaaGccqWFhpWydaqhaaWcbaGae8xVd4gabaGaeGOmaidaaaaa@3B00@ with density:

g(wi|ψi,ν)=(ψi)ν/22ν/2Γ(ν/2)wiν/2−1exp⁡(−12ψiwi)
 MathType@MTEF@5@5@+=feaafiart1ev1aaatCvAUfKttLearuWrP9MDH5MBPbIqV92AaeXatLxBI9gBaebbnrfifHhDYfgasaacH8akY=wiFfYdH8Gipec8Eeeu0xXdbba9frFj0=OqFfea0dXdd9vqai=hGuQ8kuc9pgc9s8qqaq=dirpe0xb9q8qiLsFr0=vr0=vr0dc8meaabaqaciaacaGaaeqabaqabeGadaaakeaacqWGNbWzcqGGOaakcqWG3bWDdaWgaaWcbaGaemyAaKgabeaakiabcYha8HGaciab=H8a5naaBaaaleaacqWGPbqAaeqaaOGaeiilaWIae8xVd4MaeiykaKIaeyypa0ZaaSaaaeaacqGGOaakcqWFipqEdaWgaaWcbaGaemyAaKgabeaakiabcMcaPmaaCaaaleqabaGae8xVd4Maei4la8IaeGOmaidaaaGcbaGaeGOmaiZaaWbaaSqabeaacqWF9oGBcqGGVaWlcqaIYaGmaaGccqqHtoWrcqGGOaakcqWF9oGBcqGGVaWlcqaIYaGmcqGGPaqkaaGaem4DaC3aa0baaSqaaiabdMgaPbqaaiab=17aUjabc+caViabikdaYiabgkHiTiabigdaXaaakiGbcwgaLjabcIha4jabcchaWjabcIcaOiabgkHiTmaalaaabaGaeGymaedabaGaeGOmaidaaiab=H8a5naaBaaaleaacqWGPbqAaeqaaOGaem4DaC3aaSbaaSqaaiabdMgaPbqabaGccqGGPaqkaaa@670B@

with mean *ν*/*ψ*_*i*_. Thus, the mean of the sample gene-specific variance Si2
 MathType@MTEF@5@5@+=feaafiart1ev1aaatCvAUfKttLearuWrP9MDH5MBPbIqV92AaeXatLxBI9gBaebbnrfifHhDYfgasaacH8akY=wiFfYdH8Gipec8Eeeu0xXdbba9frFj0=OqFfea0dXdd9vqai=hGuQ8kuc9pgc9s8qqaq=dirpe0xb9q8qiLsFr0=vr0=vr0dc8meaabaqaciaacaGaaeqabaqabeGadaaakeaacqWGtbWudaqhaaWcbaGaemyAaKgabaGaeGOmaidaaaaa@3055@ is 1/*ψ*_*i*_. However, the constant *ψ*_*i *_is unknown and must be estimated from the data. Instead of estimating *ψ*_*i *_separately for each gene, we consider modelling *w*_*i *_as independent observations from a finite mixture of *χ*^2 ^distributions:

g(wi|π,ψ,ν)~∑j=1kπjg(wi|ψj,ν)
 MathType@MTEF@5@5@+=feaafiart1ev1aaatCvAUfKttLearuWrP9MDH5MBPbIqV92AaeXatLxBI9gBaebbnrfifHhDYfgasaacH8akY=wiFfYdH8Gipec8Eeeu0xXdbba9frFj0=OqFfea0dXdd9vqai=hGuQ8kuc9pgc9s8qqaq=dirpe0xb9q8qiLsFr0=vr0=vr0dc8meaabaqaciaacaGaaeqabaqabeGadaaakeaacqWGNbWzcqGGOaakcqWG3bWDdaWgaaWcbaGaemyAaKgabeaakiabcYha8HGaciab=b8aWjabcYcaSiab=H8a5jabcYcaSiab=17aUjabcMcaPiabc6ha+naaqahabaGae8hWda3aaSbaaSqaaiabdQgaQbqabaGccqWGNbWzcqGGOaakcqWG3bWDdaWgaaWcbaGaemyAaKgabeaakiabcYha8jab=H8a5naaBaaaleaacqWGQbGAaeqaaOGaeiilaWIae8xVd4MaeiykaKcaleaacqWGQbGAcqGH9aqpcqaIXaqmaeaacqWGRbWAa0GaeyyeIuoaaaa@5480@

where *k *is the number of latent components (classes); *π *= (*π*_1_, ..., *π*_*k*_) are the mixture proportions with *π*_*j *_being the probability that a gene belongs to latent class *j *(*π*_1 _+ ⋯ + *π*_*k *_= 1); *ψ *= (*ψ*_1_, ..., *ψ*_*k*_) are the component-specific *χ*^2 ^distribution scaling constants and *g*(*w*_*i*_|*ψ*_*j*_, *ν*) are *χ*^2 ^distributions with the scaled constant *ψ*_*j *_being specific to component *j *and *ν *= *m*_*T *_+ *m*_*C *_- 2 is the common degrees of freedom, common for all components. The variance of the class *j *is σj2
 MathType@MTEF@5@5@+=feaafiart1ev1aaatCvAUfKttLearuWrP9MDH5MBPbIqV92AaeXatLxBI9gBaebbnrfifHhDYfgasaacH8akY=wiFfYdH8Gipec8Eeeu0xXdbba9frFj0=OqFfea0dXdd9vqai=hGuQ8kuc9pgc9s8qqaq=dirpe0xb9q8qiLsFr0=vr0=vr0dc8meaabaqaciaacaGaaeqabaqabeGadaaakeaaiiGacqWFdpWCdaqhaaWcbaGaemOAaOgabaGaeGOmaidaaaaa@30F2@ = 1/*ψ*_*j*_, the variance of all the genes in class *j*.

In mixture modelling, it is convenient to formulate the model using a missing data problem, where each observation *w*_*i *_is assumed to arise from a specific but unknown component *z*_*i *_of the mixture. The model can be written in terms of the missing data *z *= *z*_1_, ..., *z*_*g*_, otherwise known as *allocation variables*, which are assumed to be independent realizations of discrete random variables *Z*_1_, ..., *Z*_*g *_with

*Pr*(*Z*_*i *_= *j*|*π*, *ψ*, *ν*) = *π*_*j*_(*i *= 1, ..., *g*; *j *= 1, ..., *k*).

Now, conditional on *Z*_1_, ..., *Z*_*g*_, the observed data *w*_1_, ..., *w*_*g *_are independent observations from *g*(*w*_*i*_|*Z*_*i *_= *j*, *π*, *ψ*, *ν*) ~ *g*(*w*_*i*_|*ψ*_*j*_, *ν*). Furthermore, summing over all the unknown *Z*_1_, ..., *Z*_*g*_, we get g(wi|π,ψ,ν)=∑j=1kPr(Zi=j|π,ψ,ν)g(wi|Zi=j,π,ψ,ν)=∑j=1kπjg(wi|ψj,ν)
 MathType@MTEF@5@5@+=feaafiart1ev1aaatCvAUfKttLearuWrP9MDH5MBPbIqV92AaeXatLxBI9gBaebbnrfifHhDYfgasaacH8akY=wiFfYdH8Gipec8Eeeu0xXdbba9frFj0=OqFfea0dXdd9vqai=hGuQ8kuc9pgc9s8qqaq=dirpe0xb9q8qiLsFr0=vr0=vr0dc8meaabaqaciaacaGaaeqabaqabeGadaaakeaacqWGNbWzcqGGOaakcqWG3bWDdaWgaaWcbaGaemyAaKgabeaakiabcYha8HGaciab=b8aWjabcYcaSiab=H8a5jabcYcaSiab=17aUjabcMcaPiabg2da9maaqadabaacbiGae4huaaLae4NCaihaleaacqWGQbGAcqGH9aqpcqaIXaqmaeaacqWGRbWAa0GaeyyeIuoakiabcIcaOiabdQfaAnaaBaaaleaacqWGPbqAaeqaaOGaeyypa0JaemOAaOMaeiiFaWNae8hWdaNaeiilaWIae8hYdKNaeiilaWIae8xVd4MaeiykaKIaem4zaCMaeiikaGIaem4DaC3aaSbaaSqaaiabdMgaPbqabaGccqGG8baFcqWGAbGwdaWgaaWcbaGaemyAaKgabeaakiabg2da9iabdQgaQjabcYcaSiab=b8aWjabcYcaSiab=H8a5jabcYcaSiab=17aUjabcMcaPiabg2da9maaqadabaGae8hWda3aaSbaaSqaaiabdQgaQbqabaGccqWGNbWzcqGGOaakcqWG3bWDdaWgaaWcbaGaemyAaKgabeaakiabcYha8jab=H8a5naaBaaaleaacqWGQbGAaeqaaOGaeiilaWIae8xVd4MaeiykaKcaleaacqWGQbGAcqGH9aqpcqaIXaqmaeaacqWGRbWAa0GaeyyeIuoaaaa@820A@, which is just (10). We are interested in allocating gene *i *to component *j *based on its posterior classification probability *π*_*ij *_= *Pr*(*Z*_*i *_= *j*|*w*_*i*_, *π*, *ψ*, *ν*), the posterior probability of gene *i *belonging to the *j*^*th *^component. This is given as :

πij=Pr(Zi=j|wi,π,ψ,ν)∝Pr(Zi=j|π,ψ,ν)g(wi|Zi=j,π,ψ,ν).
 MathType@MTEF@5@5@+=feaafiart1ev1aaatCvAUfKttLearuWrP9MDH5MBPbIqV92AaeXatLxBI9gBaebbnrfifHhDYfgasaacH8akY=wiFfYdH8Gipec8Eeeu0xXdbba9frFj0=OqFfea0dXdd9vqai=hGuQ8kuc9pgc9s8qqaq=dirpe0xb9q8qiLsFr0=vr0=vr0dc8meaabaqaciaacaGaaeqabaqabeGadaaakeaafaqadeGabaaabaacciGae8hWda3aaSbaaSqaaiabdMgaPjabdQgaQbqabaGccqGH9aqpieGacqGFqbaucqGFYbGCcqGGOaakcqWGAbGwdaWgaaWcbaGaemyAaKgabeaakiabg2da9iabdQgaQjabcYha8jabdEha3naaBaaaleaacqWGPbqAaeqaaOGaeiilaWIae8hWdaNaeiilaWIae8hYdKNaeiilaWIae8xVd4MaeiykaKcabaGaeyyhIuRae4huaaLae4NCaiNaeiikaGIaemOwaO1aaSbaaSqaaiabdMgaPbqabaGccqGH9aqpcqWGQbGAcqGG8baFcqWFapaCcqGGSaalcqWFipqEcqGGSaalcqWF9oGBcqGGPaqkcqWGNbWzcqGGOaakcqWG3bWDdaWgaaWcbaGaemyAaKgabeaakiabcYha8jabdQfaAnaaBaaaleaacqWGPbqAaeqaaOGaeyypa0JaemOAaOMaeiilaWIae8hWdaNaeiilaWIae8hYdKNaeiilaWIae8xVd4MaeiykaKIaeiOla4caaaaa@7126@

This simplifies to:

Pr(Zi=j|wi,π,ψ,ν)=πjg(wi|ψj,ν)∑l=1kπil(wi|ψl,ν).
 MathType@MTEF@5@5@+=feaafiart1ev1aaatCvAUfKttLearuWrP9MDH5MBPbIqV92AaeXatLxBI9gBaebbnrfifHhDYfgasaacH8akY=wiFfYdH8Gipec8Eeeu0xXdbba9frFj0=OqFfea0dXdd9vqai=hGuQ8kuc9pgc9s8qqaq=dirpe0xb9q8qiLsFr0=vr0=vr0dc8meaabaqaciaacaGaaeqabaqabeGadaaakeaaieGacqWFqbaucqWFYbGCcqGGOaakcqWGAbGwdaWgaaWcbaGaemyAaKgabeaakiabg2da9iabdQgaQjabcYha8jabdEha3naaBaaaleaacqWGPbqAaeqaaOGaeiilaWccciGae4hWdaNaeiilaWIae4hYdKNaeiilaWIae4xVd4MaeiykaKIaeyypa0ZaaSaaaeaacqaHapaCdaWgaaWcbaGaemOAaOgabeaakiabdEgaNjabcIcaOiabdEha3naaBaaaleaacqWGPbqAaeqaaOGaeiiFaWNae4hYdK3aaSbaaSqaaiabdQgaQbqabaGccqGGSaalcqGF9oGBcqGGPaqkaeaadaaeWaqaaiab+b8aWnaaBaaaleaacqWGPbqAcqWGSbaBaeqaaOGaeiikaGIaem4DaC3aaSbaaSqaaiabdMgaPbqabaGccqGG8baFcqGFipqEdaWgaaWcbaGaemiBaWgabeaakiabcYcaSiab+17aUjabcMcaPaWcbaGaemiBaWMaeyypa0JaeGymaedabaGaem4AaSganiabggHiLdaaaOGaeiOla4caaa@6CEB@

The allocation of gene *i *to component *j *is based on the highest posterior probability *π*_*ij *_over all the components. Genes in the same latent class *j *share the same variance 1/*ψ*_*j*_, the mean variance of all the *n*_*j *_genes in component *j*, estimated from a larger number of replicates than purely those per gene (*n*_1 _+ … + *n*_*k *_= *n*). Parameters of the model can be estimated by the EM algorithm as shown in [17].

Quantities of interest, such as the posterior classification probabilities in (12), are estimated by *plugging-in *the point estimates π^
 MathType@MTEF@5@5@+=feaafiart1ev1aaatCvAUfKttLearuWrP9MDH5MBPbIqV92AaeXatLxBI9gBaebbnrfifHhDYfgasaacH8akY=wiFfYdH8Gipec8Eeeu0xXdbba9frFj0=OqFfea0dXdd9vqai=hGuQ8kuc9pgc9s8qqaq=dirpe0xb9q8qiLsFr0=vr0=vr0dc8meaabaqaciaacaGaaeqabaqabeGadaaakeaaiiGacuWFapaCgaqcaaaa@2E80@ and ψ^
 MathType@MTEF@5@5@+=feaafiart1ev1aaatCvAUfKttLearuWrP9MDH5MBPbIqV92AaeXatLxBI9gBaebbnrfifHhDYfgasaacH8akY=wiFfYdH8Gipec8Eeeu0xXdbba9frFj0=OqFfea0dXdd9vqai=hGuQ8kuc9pgc9s8qqaq=dirpe0xb9q8qiLsFr0=vr0=vr0dc8meaabaqaciaacaGaaeqabaqabeGadaaakeaaiiGacuWFipqEgaqcaaaa@2E91@ of *π *and *ψ*, respectively. Such plug-in estimates do not account for all the variability in estimating the model parameters and, as such, are more likely to underestimate the variance of the model parameters, which might inflate the significance levels. Aside from the problems associated with estimating the variability, the EM algorithm can sometimes have computational problems, not least in finding local maximum of the likelihood surface among several possible local maxima instead of the global maximum. In order to avoid the possibility of such problems, we propose a Bayesian hierarchical structure for the mixture model of the gene differential variance. We use an exchangeable gamma(*ς*, *τ*) prior on *ψ*_*i *_and a dirichlet(*α*_1_, ..., *α*_*k*_) prior on *π*. The hyperparameters *ς *and *τ *can be influential, and therefore in our full Bayesian analysis, these are not fixed, but given *vague *hyper-priors.

## Competing interests

The funding bodies were not involved at all at any stage of the manuscript preparation. There are no competing interests and the authors have nothing to declare.

## Authors' contributions

SOMM and MSG conceived the original idea. SOMM researched, developed the methodology, designed and fitted the Bayesian model, interpreted the analyses, and wrote the first and subsequent drafts. REW extracted and prepared the data and ran the EM algorithm macro supplied by [17]. MSG reviewed and commented on the drafts. All authors read and approved the final version.
